# Testing Thermostatic Bath End-Scale Stability for Calibration Performance with a Multiple-Sensor Ensemble Using ARIMA, Temporal Stochastics and a Quantum Walker Algorithm

**DOI:** 10.3390/s23042267

**Published:** 2023-02-17

**Authors:** George Besseris

**Affiliations:** Department of Mechanical Engineering, The University of West Attica, 12241 Egaleo, Attica, Greece; besseris@uniwa.gr

**Keywords:** temperature calibration, sensor ensemble, thermostatic bath, ARIMA, quantum walker, uniformity, stability, stationarity, normality

## Abstract

Thermostatic bath calibration performance is usually checked for uniformity and stability to serve a wide range of industrial applications. Particularly challenging is the assessment at the limiting specification ends where the sensor system may be less effective in achieving consistency. An ensemble of eight sensors is used to test temperature measurement stability at various topological locations in a thermostatic bath (antifreeze) fluid at −20 °C. Eight streaks of temperature data were collected, and the resulting time-series were processed for normality, stationarity, and independence and identical distribution by employing regular statistical inference methods. Moreover, they were evaluated for autoregressive patterns and other underlying trends using classical Auto-Regressive Integrated Moving Average (ARIMA) modeling. In contrast, a continuous-time quantum walker algorithm was implemented, using an available R-package, in order to test the behavior of the fitted coefficients on the probabilistic node transitions of the temperature time series dataset. Tracking the network sequence for persistence and hierarchical mode strength was the objective. The quantum walker approach favoring a network probabilistic framework was posited as a faster way to arrive at simultaneous instability quantifications for all the examined time-series. The quantum walker algorithm may furnish expedient modal information in comparison to the classical ARIMA modeling and in conjunction with several popular stochastic analyzers of time-series stationarity, normality, and data sequence independence of temperature end-of-scale calibration datasets, which are investigated for temporal consistency.

## 1. Introduction

From new-age industrial transformation to future community development, the Internet of Things (IoT) plays an important role in propelling the green economy forward and achieving worldwide sustainability goals [1,2,3,4]. From the need to ensure global food sufficiency and security to managing sustainable construction in order to accommodate an increasing world population, IoT takes center stage in the realization of the fourth industrial revolution [5,6]. To achieve the prudent use of resources, smart technologies are critical to monitoring and controlling the modern digital infrastructure. Since the contemporary philosophy of living progresses on a data-centric framework, sensors are the building blocks of information generation, because they convert experiential conditions to valuable data. Smart sensors are not only anticipated to be indispensable to the “Industry 4.0” enterprise, but also to daily life [7,8,9]. By 2050, an expected 85% of the world population is projected to live in resource-efficient and socially inclusive urban areas [10]. Consequently, high quality of life in a green economy will only be possible by effectively orchestrating a great wealth of fused information, which will be created from an immense number of smart sensor networks. Smart sensors are already in demand in diverse areas of utilization, including modern energy systems, energy-smart buildings, water resource management in urban and farming applications, healthcare operations, as well as in environmental air-quality monitoring and processed food improvement [11,12,13,14,15,16,17,18,19].

As large networks of sensor systems are bound to process and distribute an enormous number of data streams, new challenges emerge in ensuring data fidelity and stability to the data analysis channels [20]. Consequently, an obvious intricacy stems from the fact that, in the future, computing will rely on an enormous number of deployed sensors that, in conjunction with their enormous task of incessantly feeding data to a broad range of processors, will create vast data volumes which will be delivered at high velocities. Thus, the stability of the sensors at the endpoints of the calibration scale is imperative at the inception of the data generation process to ensure overall reliability in the data streams. 

Temperature is a fundamental quantity in nature. Accurate temperature readings may be pivotal in invasive engineering measurements and wherever there is a need to remotely track temperature recordings in a medium, such as when a fluid is confined in a container or a tank. Temperature sensors may experience issues of accuracy, sensor (unit-to-unit) variation, medium heterogeneity, and thermal fluctuations during calibration [21,22]. To accomplish thermostatic bath temperature consistency, measurements may involve simultaneous monitoring from a group of sensors located at preset depths and distances. Thus, there may be a topological effect that contributes to the variability of measured temperature.

Particularly important is the sensor calibration at the expected temperature operating ends, since measurement stability must be demonstrated for the specified tolerances. To study the stability of temperature readings, time series analysis is usually employed as part of the ordinary device measurement analysis procedure. A confident calibration procedure relies on the repeatability and reproducibility of the measurements [23,24]. A gage R&R study will check the presence of linearity and bias in the recordings, run charts will allow inspecting for data independence, while histograms and statistical tests will provide the significance of the observed measurement errors [25,26]. If the gage proves to be stable, temperature variation is compared to the prespecified tolerance, and the sensor capability will be estimated [27]. Fundamental time series analysis may also be applied to establish significance of stationarity which is presumed for process control [28]. Modelling autoregressive and moving-average parameters of the data streams should next be examined through residual analysis tests.

Tracking medium temperature patterns using an ensemble of dispersed sensors at various topological locations in a fluid medium may be a complex process; it may be modulated by several sources of uncertainty. The selected topological configuration of the sensors may be susceptible to fluctuations due to medium heterogeneity, and further exacerbated by the unit-to-unit variation of the participating sensors in the ensemble. The overall sensor system study may become quite complex as many opportunities for uncertainty intrusions continue to appear, even during the analysis phase. For example, there are various types of statistical tests to select from and their predictions may vary among them. Even the optimal presetting of the level of significance for a specific study may be debated [29,30,31,32]. Perhaps the new statistics that have been suggested to improve the scientific process would relieve the limited role of the traditional statistical inference in the difficult task of formalizing the course of the knowledge development [33,34]. The challenge remains as long as the stochastic hierarchy is still to be discovered; effects of lesser probabilistic impact should be dismissed [35] Therefore, it is up to the methodological decisions to provide the level of universality of a hypothesis via the *modus tollens* logic [36]. 

The purpose of this work is to introduce a simplistic approach to quickly determine a potential inherent instability among several streaks of temperature measurements that have been generated from an ensemble of (same-type) sensors. The sensor group is assumed to be situated in a fluid medium in a calibration process and collected measurements are limited at temperature operating range ends. New diagnostics insight in this endeavor will come from considering the implementation of continuous-time quantum random walks [37,38,39,40,41,42,43,44,45,46], in lieu of treating the collected dataset with a classical statistical process control theory [25], and/or a customary time-series analysis procedure [28]. Time series analysis evaluates the stationarity status of the streaking observations, thus permitting the estimations of autoregression and moving-average parameter comparisons among the assembled sensor units. The motivation for implementing quantum random walks is three-fold: (1) quantum theory has a universally ‘grassroots’ stochastic framework [47], (2) the computational universality of quantum walks is also known [40], and (3) the natural emergence feature of randomness and the arrow of time in quantum walk stochastics [44]. This is essential because the random walk and the quantum walk exhibit different behaviors [48]. 

Adopting quantum walk stochastics may be redeemed in gained computational simplicity. It enables the comparison of the stability assessments across the multiple sequences of temperature measurements by uniformly fingerprinting the data streaks in terms of network node transitions by a quantum walker. Thus, the sensor ensemble stability performance is only needed to be contrasted against its predicted model modes alone. It is this pivotal point that the novelty of this empirical work focuses on. Additionally, the proposed approach takes on a task-specific quick-turnaround research study that is understood to be favored due to its designed-in data frugality [49]. This tactic allows further reduction of the required types of extracted information as well as its relative volume in order to arrive to meaningful comparisons across the multiple time-series data. The practical aspect of the presentation relies on being readily able to directly use an open and diverse software platform to analyze the examined time series dataset, such as the toolbox of the R-language freeware [50,51,52]. The adoption of open data science tools has been encouraged because they promote faster and more credible results in the knowledge discovery cycle [53]. The data in the case study have been partially drawn from a research project on investigating temperature calibration performances. 

An extra advantage of this work is that it compares the ordinary time-series analysis outcomes from classical statistical process control monitoring with a continuous-time quantum-walk algorithmic model-fitting procedure, which aspires to incorporate disparate kinds of information views from both worlds and, hence, to furnish a more holistic data treatment outlook [54]. Since the distribution homogeneity in the assembled data streaks is not guaranteed, fingerprinting the underlying uncertainty may only be depicted by employing various visualization techniques in hoping to uncover fine details in the examined stochastic structures [55,56]. 

There is no previous work on this subject to the best of the author’s knowledge. The novel contribution is couched on the introduction of a quantum-walker time-series analysis to detect modal stability across the data sequence for the calibrating sensor-ensemble configuration, at the limiting sensor end-scale temperature boundary. From a computational implementation standpoint, the quantum-walker ‘sequencer’ is preferred over the more widely disseminated tree-based ensemble learning or cutting-edge deep-learning algorithms, because the quantum-walk solver is not susceptible to prediction attenuation by several “internal clockwork” hyperparameters, which otherwise cannot be ignored. Moreover, the applied stochastic analysis employs a combination of Auto-Regressive Integrated Moving Average (ARIMA) theory [28] and other time-series property-oriented statistical treatments in normality, stationarity, and in checking the identical-and-independent data-sequence distribution; they provide a framework for verifying the time-series instabilities at the temperature calibration endpoint. 

Next, this paper presents a two-part Methodology section, in which a traditional treatment of statistical process control and time series methods are implemented such that to offer regular inference predictions. The deployed quantum random walk solver will also be described along with the required computational amenities. In the Results section, ample data-centric time-series manipulation work will be provided to facilitate the comprehension of diagnosing critical stability issues, while attempting to calibrate a particular configuration of an ensemble of temperature sensors on the specification limits in a fluid medium. Finally, in the Conclusions section the main findings are restated and future work is suggested.

## 2. Materials and Methods

### 2.1. Theoretical Aspects in the Methodological Developments

#### 2.1.1. The Statistical Time Series Screening Assisted by ARIMA Modeling

This study treats the collected temperature observations as multiple-input time series data that have been generated from several pre-arranged (fixed) locations in a fluid medium. Two approaches are applied in determining measurement stability in order to provide the necessitated confidence in assessing the performance of the calibration process. The first approach follows a customary procedure to investigate the behavior of the time series. Thus, the implemented methods involve evaluations of the data normality, as well as assessing the time-series stationarity and its invertibility status. The invertibility condition is important as estimations of the residuals reflect the true random fluctuations. 

Data symmetry is also examined in parallel for skewness and kurtosis. Visual illustration of the relative time series groupings is facilitated by using at least two comparative graphical screenings. Simultaneous plotting of the time series from all collection location points is depicted in regular boxplot formations alongside the more specialized violin graphs. The ARIMA theory [28] is employed to uncover any potential co-influences as detected from autoregression (AR) and moving average (MA) stochastic evaluations. The AR terms are the lags of the stationarized series. The MA terms are the lags of the forecast uncertainty. ARIMA is used generically in case that differencing might be applicable in order to render the integrated time series stationary. Statistically, a time series is stationary if the central tendency, the variation, and the assorted autocorrelations demonstrate firmness in time; neither trends nor heteroscedasticity are disposed to agitate the time-series fluctuation rhythm at any segment. All considered time series are checked for unit-root presence and, hence, potentially hinting at an underlying linear trend. The absolute values for all AR roots must be less than 1 for the model to be stationary. It is imperative to demonstrate that all monitored signals produce a reliable homogeneous status for the examined boundary temperature specification according to the adopted calibration scheme. The tactic of including concomitant inferences from classical statistical tests along with the results from the ARIMA predictions might aid in detecting any cancellations between the AR and MA terms in the integrated modeling endeavor. 

Model identification of the AR and MA parameters is attained by obtaining the plots of the Auto Correlation function (ACF) and the Partial Auto Correlation function (PACF). The statistical significances of the coefficient estimate of the AR and MA are also estimated and, if they are found comparable in magnitude, the AR and MA model coefficients are examined for self-cancellation. Residual diagnostics from the ACF and PACF screenings suggest the hierarchical strong coefficients that dictate the time series pattern. We define the value of a process time series to be: *y_t_*, *y_t_*_−1_, *y_t_*_−2_…; for equally spaced times: *t*, *t* − 1, *t* − 2, …. The control-and-forecast equation, $\bar{y_{t}}$, is succinctly written with respect to the *p*-lagged values of the AR terms in the *y_t_* time series, along with incorporating the *q*-lagged errors of the MA terms and, in adjustment by a constant term, *μ* as:

$$\bar{y_{t}}=\mu +\overset{p}{\underset{i=1}{\sum }}\varphi_{i}y_{t-i}-\overset{q}{\underset{j=1}{\sum }}\theta_{j}e_{t-j}$$

where $\varphi_{i}$ (1 ≤ i ≤ *p*) and $\theta_{j}$ (1 ≤ j ≤ *q*) are the corresponding regression coefficients for the AR and MA terms. Differencing (Δ*z_t_*) is used in the ARIMA(*p,d,q*) model fitting attempt in case it is deemed that the time series is necessitated to be stationarized. Ostensibly, if no differencing is needed, then, *d* = 0 (*z_t_ =* $y_{t}$), otherwise:

$$\begin{array}{c} \mathrm{For}\;d=1:\;\Delta z_{t}=z_{t}-z_{t-1}\;=y_{t}\\ \mathrm{For}\;d=2:\;\Delta z_{t}\;-\;\Delta z_{t-1}=(z_{t}-z_{t-1})\;-\;(z_{t-1}\;-\;z_{t-1})=y_{t}\;\mathrm{and}\;\mathrm{so}\;\mathrm{forth} \end{array}$$

#### 2.1.2. Time Series Mode Screening Using a Quantum Walker

Quantum walk algorithms have become a research subject of great interest in information processing studies due to their effectiveness in applications that involve pattern recognition tasks. A continuous quantum walk algorithm will be employed in this work. It is a more general option as its probabilistic evolution operator does not have to be confined to discrete time steps. The model involves a stochastic walker and the Hamiltonian evolution operator. Therefore, the Schrodinger equation dictates the pacing of the solver. The motivation to replace the diffusive transport mechanism that directs a classical random walker is justified by addressing the ballistic spread advantage that the quantum mechanical wave function offers in an evolving solution. The quantum walk solver accelerates due to its variance dependence on the step number, which enjoys quadratic gain over the classical random walker [57]. Moreover, due to the exponential localization of the quantum wave in static disorder conditions, the spread stabilizes and the variance becomes constant. 

The quantum walker is used to track down random temperature fluctuation tendencies that may propel an observation to exit the measurement specification zone. The walker is assigned a group of probabilities that will exit, sooner or later, any node (time point in a time series) on the network (time series). The theoretical development requires an adjacency matrix A that interconnects nodes and edges. If the base state |j〉 is defined in the Hilbert space H, the evolution of state vector $|\varphi \left(t_{k}\right)\rangle$ of the quantum walker at a given time $t_{k}\;$is defined as:

$$|\varphi \left(t_{k}\right)\rangle =\sum a_{j}\left(t_{k}\right)|j\rangle \;\;\mathrm{with}\;\left|a_{j}\left(t_{k}\right)\right|\;\in \left[0,1\right]$$

And it progresses according to the unitary transformation:

$$\frac{d}{dt}\left|\varphi \left(t_{k}\right)\rangle =i\;A\right|\varphi \left(t_{k}\right)\rangle \;\mathrm{and}\;\mathrm{hence}\;\left|\varphi \left(t_{k}\right)\rangle =e^{-iA}\right|j\rangle$$

The probability of a quantum walker on a node, for the base state |j〉 at a given time $t_{k},\;$is defined as:

$$p\left(t_{k},\;|j\rangle \right)={\left|a_{j}\left(t_{k}\right)\right|}^{2}\;\mathrm{with}\;\sum {\left|a_{j}\left(t_{k}\right)\right|}^{2}=1$$

Therefore, the probability for moving from node *n* to node *m* is:

$$p_{nm}\left(t_{k}\right)={\left|\left|t\right||\varphi \left(t_{k}\right)\rangle \right|}^{2}\;\mathrm{where}\;\left|t\right|\;\mathrm{is}\;a\;\mathrm{state}\;\mathrm{transition}\;\mathrm{matrix}$$

The above quantum walk computations are carried out on the adjacency matrix A:
0100000010100000010100000010100000010100000010100000010100000010
Using the Cayley–Hamilton theorem, a system of equations is solved to obtain the coefficients of approximating the time evolution operator. 

### 2.2. The Case Study for Testing Temperature Stability with a Sensor Ensemble

The case study exemplifies tracing temperature-time trends in raw sequenced datasets, which are borrowed from a very recent thermostatic bath calibration project that explored issues of uniformity and stability at different limiting temperature specification ranges [58]. The dataset collection scheme assumed an eight-sensor configuration for temperature measurements as published in Zeng et al. [59]. The experiments were conducted, at an accredited laboratory, by ISO 17025 (Val Electronic, Greece) which specializes in calibrating a wide range of thermometrical instruments and devices. The dataset was specifically selected to investigate the calibration performance at the lowest specification limit of −20 °C. As described in the report [58], the examined bath fluid in the apparatus was ethylene glycol (antifreeze liquid) contained in a stainless-steel thermostatic bath connected to a data logger which completed the thermometric process. The indicator resolution and accuracy were 0.01 °C and ±0.05 °C, respectively. 

In brief, eight measurements were collected from eight different topological locations in the bath fluid. The sensor probes were platinum-based resistance thermometers (PT 100). The rate of data collection was paced at 5 s and the trials were concluded after 22 min. Therefore, eight temperature time-series datasets were formed, each comprising of 256 observations. Receiving information from a multiple monitored temperature points offers more opportunities to detect out-of-specification measurements and non-random fluctuations as there are several sources of instability in such a physical system. Sensor unit instabilities, medium fluid heterogeneity, temperature non-homogeneity at different depths in the thermostatic bath, and random temperature discrepancies at various distances from the center line (on the same plane) could cause some observations to manifest divergent behaviors and outlier tendencies. 

### 2.3. The Methodological Outline

**Scheme 1:**
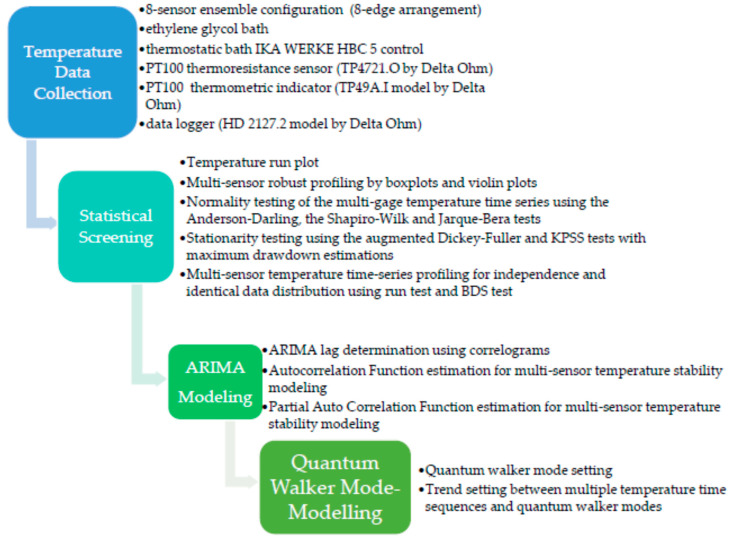
Detailed flowchart of the methodological steps.

The methodology is summarized in the following steps (Scheme 1):

(1) Determine the type of physical measurements that must be performed in the calibration gage limits.
(2) Select the appropriate testing medium.
(3) Determine a convenient size for the sensor ensemble and spatially arrange them in the testing medium.
(4) Collect and record the data streaks from each sensor unit in the ensemble.
(5) Compare time-series data distributions by conducting robust screening through ordinary boxplot depictions [60]. Moreover, supplement the information from the parallel visuals using violin plots to portray the local density trace properties in more detail [61].
(6) Perform normality tests on individual sensor (time series) observations, by implementing techniques like the Anderson–Darling test [62], the Shapiro–Wilk test [63], and the Jarque–Bera test [64].
(7) Perform time-series stationarity assessments by employing the augmented Dickey–Fuller test [65] and the KPSS test [66], while complementing the resulting inference outcomes with the information loss evaluation as it is quantified by the maximum drawdown estimation.
(8) Assess the randomness of the time sequences and whether they are independent and identically distributed by using the run test [67] and the BDS test [68,69].
(9) Assess the ARIMA modeling results of the individual temperature data streaks by considering the lags of the stationarized series (AR terms) and the lags of the forecast errors (MA terms) [28].
(10) Evaluate the significance of the AR and MA coefficients.
(11) Index the individual time series modes using a continuous-time quantum random walk algorithm and check for proper model fitting by testing the behavior of the residuals on a Q-Q plot.
(12) Retain and compare the characteristic number of modes among the individual temperature data streaks. Finally, inspect the selected cut of modes for similarities among different time series and test the time sequences according to the Akaike information criterion [70].

### 2.4. Computational Aids

The computational and graphical work was carried out on the statistical freeware platform R (v. 4.1.3) [52]. Regular boxplot screening of the multiple temperature data streaks was performed using the ‘boxplot ()’ function from the R-package ‘graphics()’ (v. 4.1.3). Correspondingly, the specialized violin-plot screening was prepared by employing the R-package ‘vioplot()’ (v.0.3.7). The normality of the eight-sensor time-series data was examined using three approaches: (1) the goodness-of-fit procedures of the Anderson–Darling test, (2) the Shapiro–Wilk test, and (3) the Jarque–Bera test, by implementing the functions ‘ad.test()’ (R-package ‘goftest()’ (v.1.2-3)), ‘shapiro.test()’ (R-package ‘stats()’ (v.4.1.3)), and ‘jarque.bera.test()’ (R-package ‘tseries()’ (v.0.10-51)), respectively. To test the individual time series for stationarity, the augmented Dickey–Fuller test and the KPSS test were employed using the functions ‘adf.test()’ (R-package ‘tseries()’ (v.0.10-51)) and ‘kpss.test()’ (R-package ‘tseries()’ (v.0.10-51)), respectively. 

The maximum loss on the individual time series dataset was computed using the function ‘maxdrawdown()’ (R-package ‘tseries()’ (v.0.10-51)). The runs test for randomness was conducted by employing the function ‘RunsTest()’ (R-package ‘DescTools()’ (v.0.99.45)). Moreover, the function ‘bds.test()’ (R-package ‘tseries()’ (v.0.10-51)) was also implemented to test whether the random temperature sequences were independent and identically distributed. To fit the ARIMA models in order to diagnose regressed autocorrelated errors, the function ‘sarima()’ (R-package ‘astsa()’ (v.1.15)) was deployed. Lag order information from the ACF and PACF graphical results were fed to function ‘arima()’ (R-package ‘stats’(v.4.1.3)) to evaluate the statistical significance of the AR and MA coefficients and to obtain the residual analysis diagnostics.

The R-package ‘QWDAP’ (v.1.1.17), which is suitable for alternative data-centric engineering analysis and prediction, was deployed due to its capability to model graph-associated time series, using a path finder as a continuous-time quantum walker. The function ‘qwdap.qwalk()’ was initialized by introducing an 8 × 8 adjacency matrix and their accompanying scaling factors. A stepwise linear regression analysis fitted the temperature time series profile to the modes of the quantum walk procedure using the function ‘qwdap.swr()’. The hierarchical mode selection, which elicits similar behavior among the different temperature time series profiles according to the quantum-walk mode finder, was accomplished using the function ‘qwdap.sws()’. 

## 3. Results

### 3.1. Statistical Analysis of the Sensor Ensemble Time Series Data

#### 3.1.1. Basic Inferential Testing of the Sensor Ensemble Temperature Data Streaks

**Figure 1:**
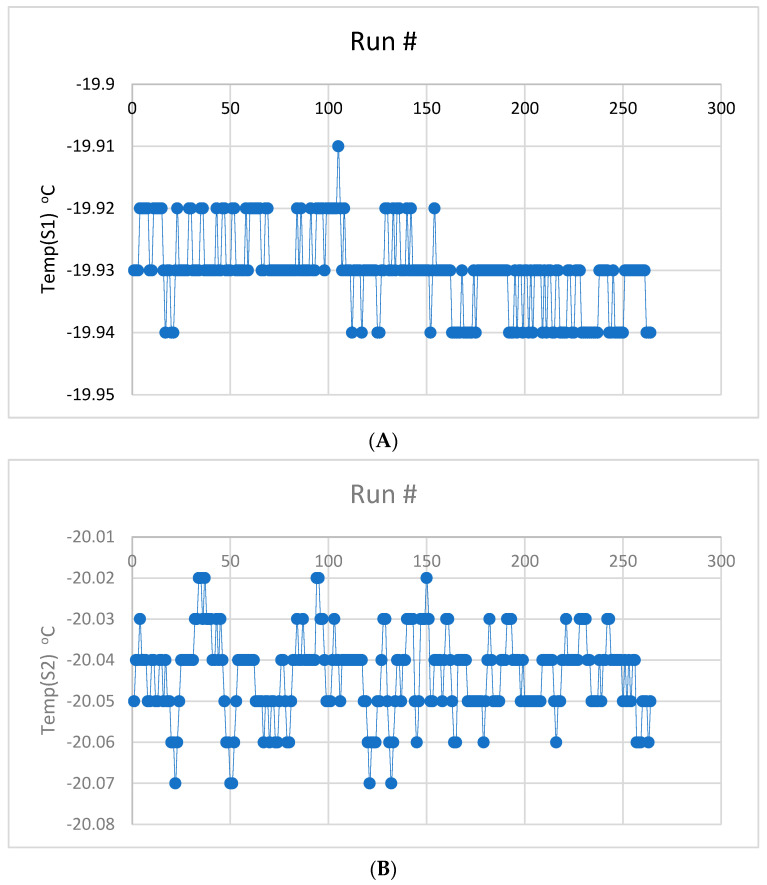

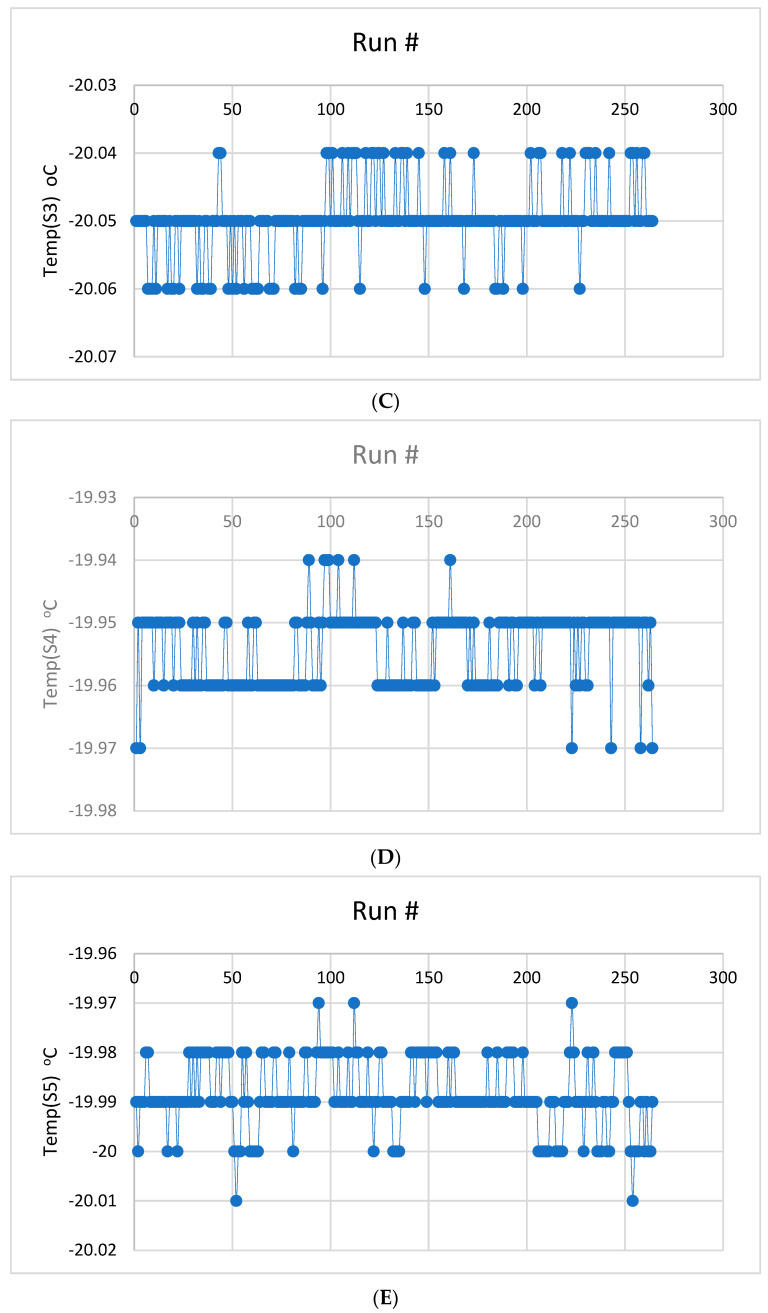

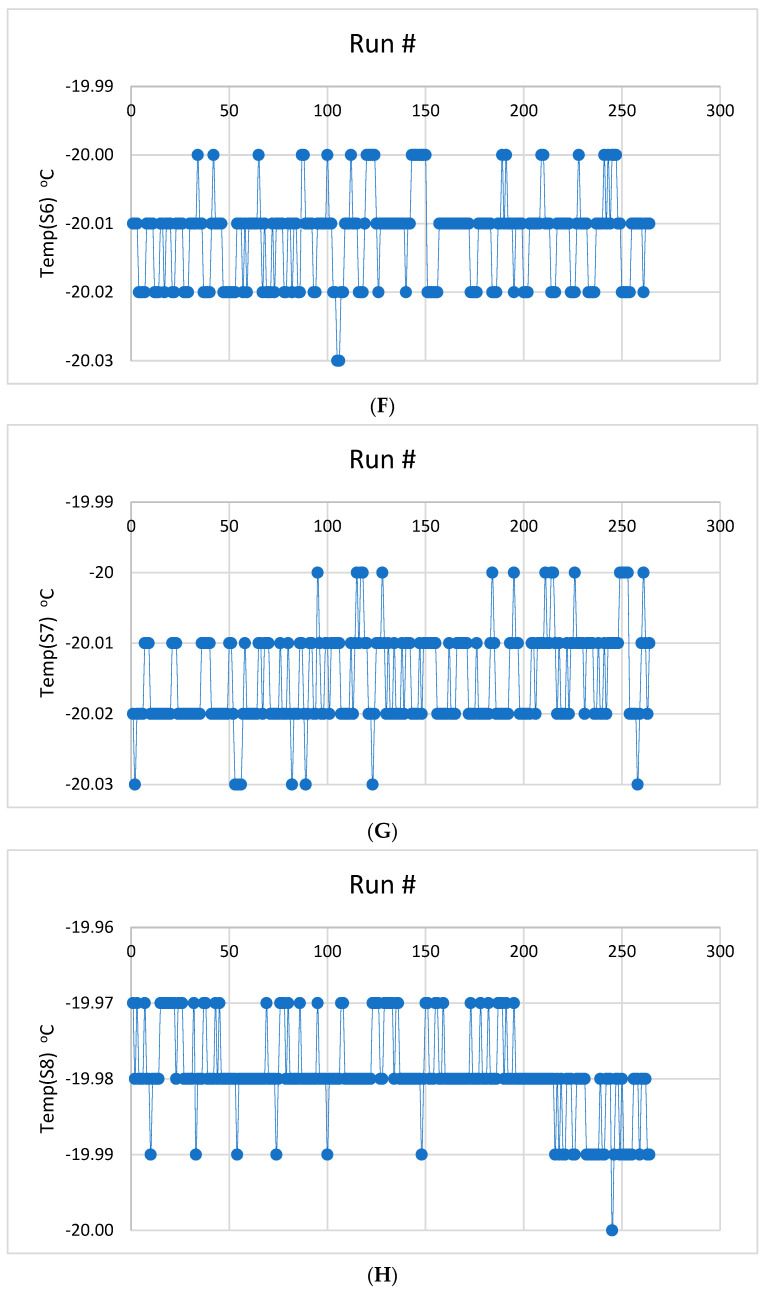
Line graphs with data points for the eight temperature time series with increasing sensor identification numbers: (**A**–**H**) temperature plots.

**Figure 2:**
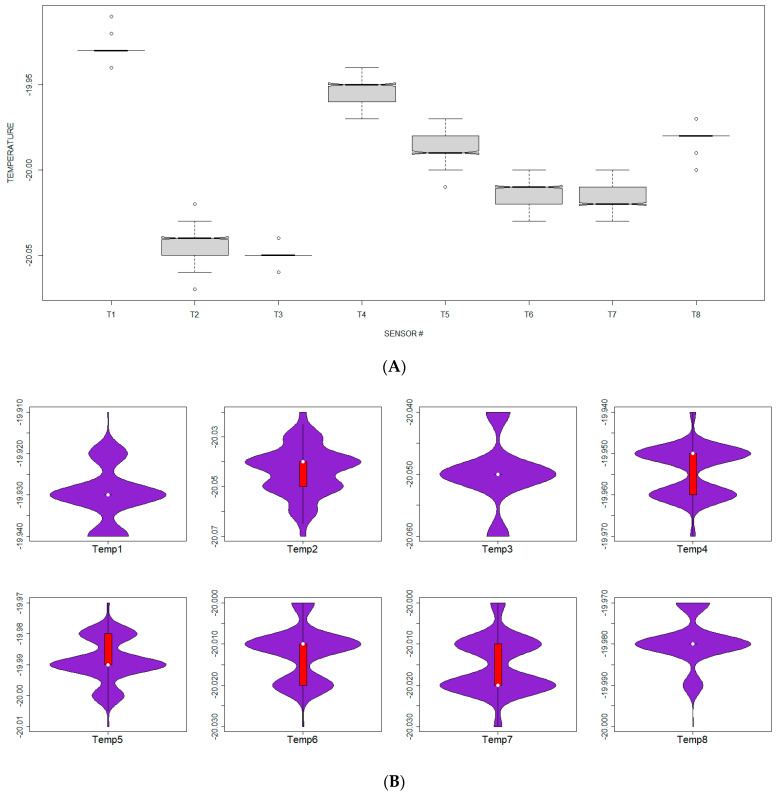
Boxplot (**A**) and violin plot (**B**) screenings of the temperature data (in °C) from all eight sensors.

The temporal visualizations of the eight time series is shown in Figure 1 in simple (uncurated) line charts that only include the raw data points. From the incurring data trends, it is noticed that the rate of change of the temperature-time profiles are all different. Even though the frequency density of the data points and their accompanying spread tendencies are distinct for each set of sensor measurements, it seems that the individual datasets may be categorized to four ‘quantized’ levels: (1) plot C (3-level), (2) plots A, D, F, G, and H (4-level), (3) plot E (5-level), and (4) plot B (6-level). The four data-point grouping occurs more often, in five out of the eight datasets. The temperature data streaks from the eight-sensor ensemble are easily summarized and compared by attempting a box-plot screening. In Figure 2A, it is easily observed that there is a variability among the eight sensors with respect to their central tendencies and their dispersion propensities. Clearly, the sensors coded #1, 2, and 3 produce measurements that may lie outside the specification range of ±0.05 °C. Sensors #1, 2, and 8 appear to generate only outlier observations, in the absence of any visible data variation. Sensor #1 produces all temperature data points above the upper specification limit, while the temperature median value of sensor #3 is located on the lower specification limit. 

The measurement variations from sensors #2, 4, 5, 6, and 7 possess a similar magnitude but their temperature median values display a strong asymmetric tendency favoring no particular side. Not all sensors generate extremities or outlier data points: sensors #4, 6, and 7 appear to be more stable. Based on the location of the sensors in the calibration configuration, there is significantly different data grouping. The temperature readings from the four sensors, which are situated on the (same) top plane arrangement in the bath fluid (they were coded as #1–4), display a distinctive instability that persistently lingers around either of the two temperature specification endpoints. 

On the contrary, the bottom (four-sensor) arrangement is stable and well contained within the temperature specification boundaries.

This might imply that fluid uniformity was achieved during the data collection process in the bottom fluid layers of the bath. It is dubious as to what the upper sensor arrangement indicates, especially when considering the opposing but narrow assemblage of temperature readings at sensor units #1 and 3. In view of the fact that the boxplots assume symmetry to construct the whisker marks, along with the normality condition to adjust their reach, combined skewness and kurtosis manifestations may be conveniently probed by drawing the respective violin plots. Accordingly, the violin-plot screening (Figure 2B) surely provides a more detailed fingerprinting congruent to the dispersive properties of the data distributions.

There is significant variability among the contours of the distribution silhouettes for the entire temperature serial dataset. 

As they are smoothed by the rotated kernel density estimations, the probability fluctuation motifs depict multifarious heterogeneous behaviors, which are projected by eight distinct data generating mechanisms. Shapes are portrayed to be multimodal. They vary on the number and size of protuberances as well as on their peak positions and relative amplitudes. The rhythm of the modulated temperature profiles does not appear to be distributionally correlated on a one-to-one basis, i.e., contrasting between two different data sequences.

Based on the comments on the visual-descriptive analysis of the temperature serial datasets above, the typical statistical inference commences with the normality testing of the eight temperature data sequences. In Table 1, the results from the three ordinary goodness-of-fit methods are listed. Departures from normality, in terms of their estimated statistical significances, have been designated for the Anderson–Darling test, the Shapiro–Wilk test, and the Jarque–Bera test. The Anderson–Darling test outcomes are in close agreement with those from the Shapiro–Wilk test: all eight data sequences stochastically exhibit non-normal behaviors. Furthermore, the Jarque–Bera test outcomes offer a disparate view on establishing uniform goodness-of-fit performances. This might be expected as the Jarque–Bera technique synchronously consolidates direct information from the joint influence evaluation of the serial sample skewness and the kurtosis. It is statistically inferred that only the temperature dataset which originates from sensor unit #7 does not adhere to the joint (serial sample) normality hypothesis; the remaining seven datasets abide to the normality assumption. 

Since there is such a conflict in the predictions of the normality status of the eight time series, a stationarity screening may provide potential evidence about the sources of the observed discrepancies. In Table 2, the augmented Dickey–Fuller test outcomes suggest the absence of unit roots and they allocate stationarity to all temperature serial samples, except to the one identified as the data sequence collected by sensor unit #4. To examine whether any of the time series is stationary around a deterministic trend, the KPSS test was applied to the dataset and the results are listed on Table 2. It is found that the temperature time series #2, 4, 5, and 6 may be characterized as trend-stationary, while the remaining four time series are suspected to contain a unit root. How enduring this influence is on the sample mean values should be analyzed using the more specialized method ARIMA. From the same table, it is noted that the maximum drawdown estimates are tightly maintained: they narrowly vary from 0.02 to 0.05 °C. 

Finally, the eight data sequences were treated with the runs test and the BDS test in order to assess whether the inspected serial elements may be classified as independently and identically distributed. According to the runs test results (Table 3), all estimated significance values agree: none of the tested time series may be deemed random (*p* < 0.01). Similar inference is obtained from the BDS test, excluding the behavior of time series #3. Particularly for the case of time series #3, in three out of the four appraised threshold distance (ε) values, the statistical test does not detect any departure from randomness. It is only at a value of ε set to two standard deviations that it is found to be statistically significant (*p* < 0.001).

#### 3.1.2. Autoregression and Moving Average Parameter Screening

**Figure 3:**
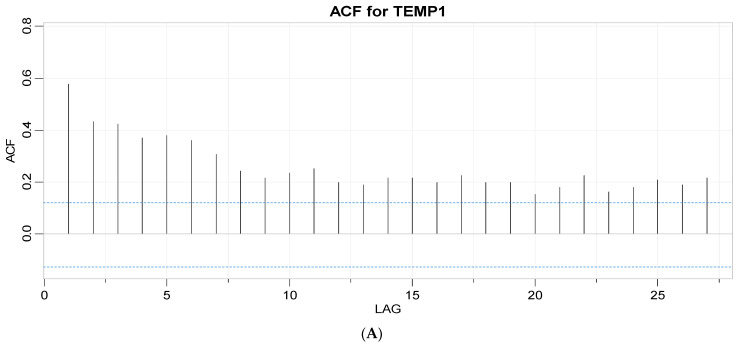

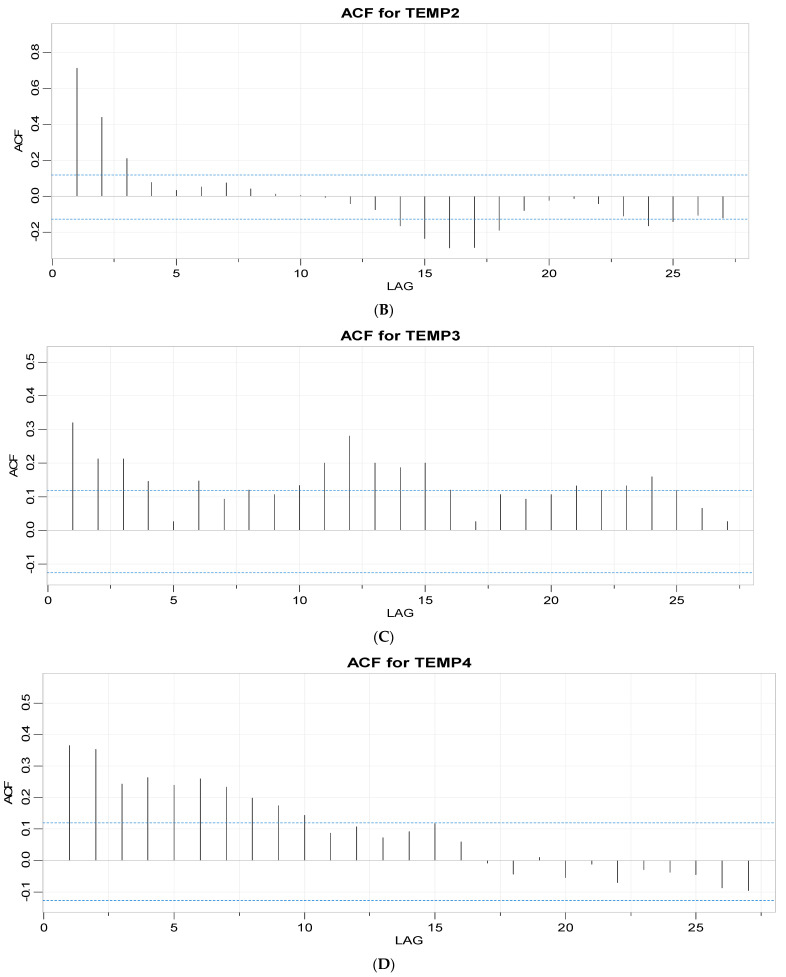

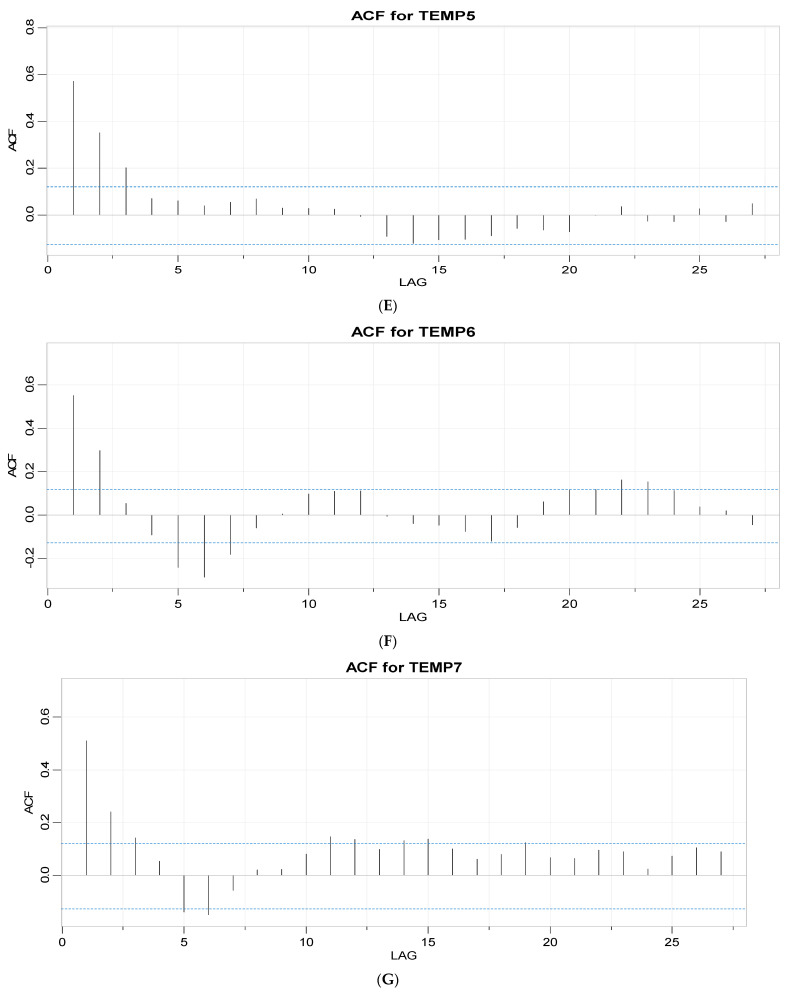

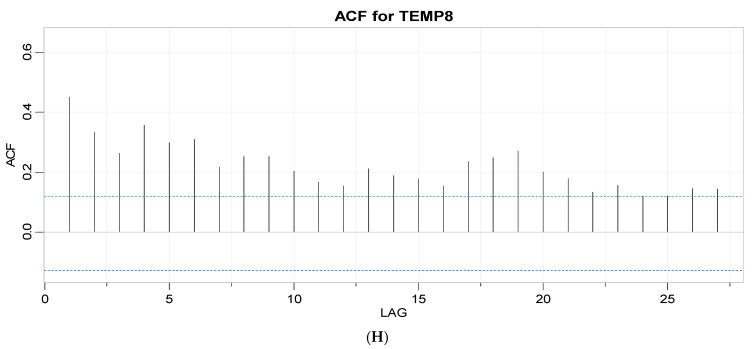
Correlograms for the eight time series: (**A**–**H**) temperature ACF plots.

**Figure 4:**
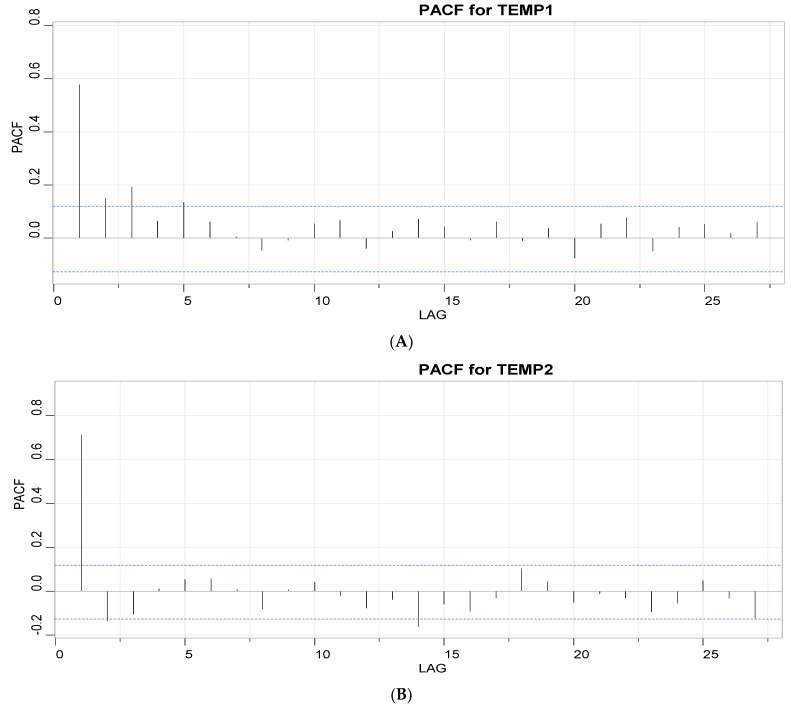

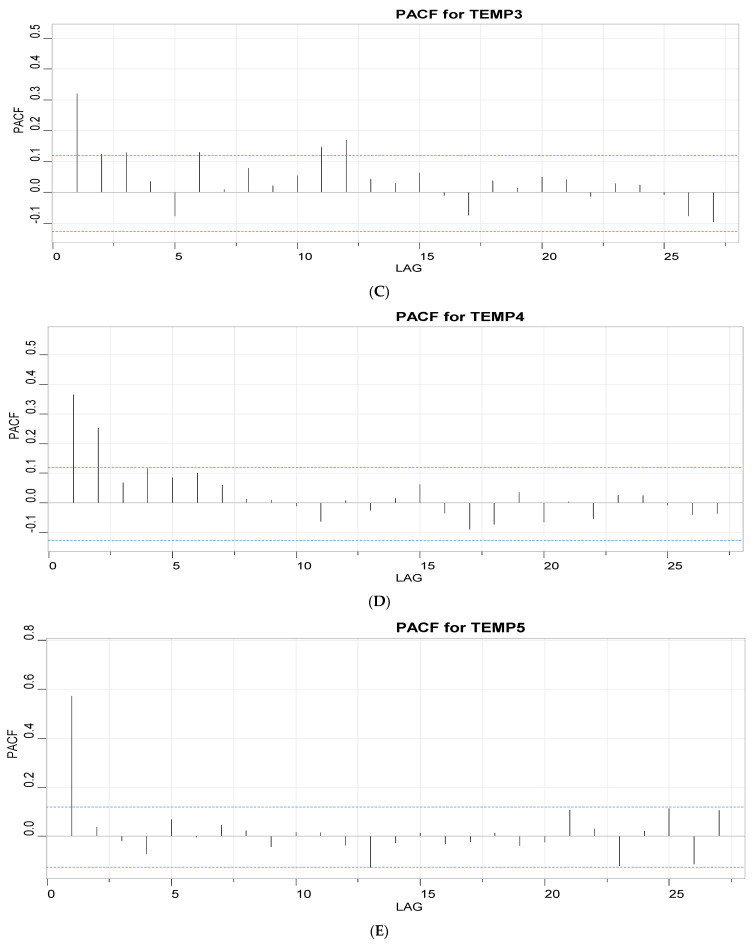

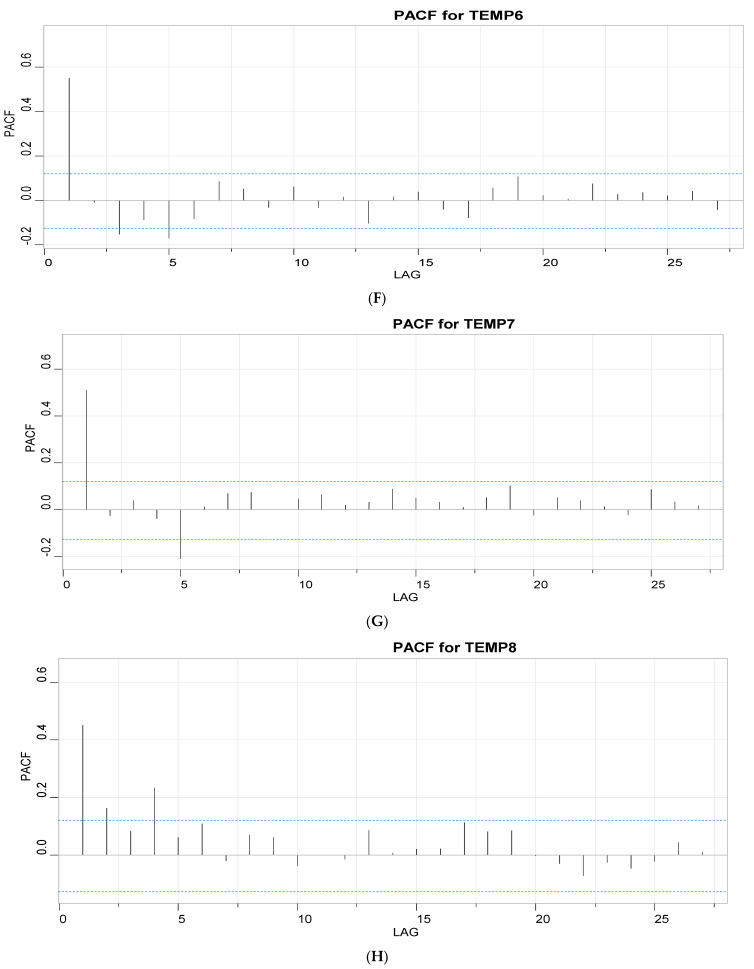
Partial autoregression correlograms for the eight time series: (**A**–**H**) temperature PACF plots.

The spectrum of the respective autocorrelation functions for the eight temperature time series is shown in Figure 3 in individual ACF correlograms. The challenge here is not to discover a single forecasting function, with exceptional goodness-of-fit performance, but in predicting the future behavior for each temporal sequence. Instead, the extent of disparity that is evidenced in the initial patterns of the eight ACF lag signatures is sought. Cogent tracking of the ACF-lag spectrum manifestations is pivotal because the order and the pattern of the lags determine whether there is a need for stationarizing the temperature profile by differencing. Additionally, the effect of the MA coefficients in the ARIMA modelling is quantitatively justified. In Figure 3, it is noted that there is significant variation among the spans of lags. There are several candidate lag values to be delved into the next phase of the ARIMA data processing. None of the eight ACF correlograms could be viewed as matching the sequence pattern of the others. For example, in Figure 3A, lag ordering is exhausted to the package’s default limit of 27. All lags positively affect the ACF estimations. Also, taking in account the PACF spectrum (Figure 4A), the positively dominate spike at the first lag suggests that time series #1 should be differenced. It is diagnosed as nonstationary in disagreement to the augmented Dickey–Fuller test outcomes (Table 2) that had previously inferred its statistical stationarity. 

Moreover, it is in closer agreement to the respective KPSS test outcome, i.e., suspecting the presence of a unit root. It may be examined for a constant average trend (first order differencing). If, upon inspection, the first order differencing causes the spectrum patterns of ACF and PACF to persist, it would be prudent to contemplate a time-varying trend (second order differencing). Besides, in Figure 3B, the spectrum spikes are alternating to negative ACF values at a lag value as high as 24, even though the first lag along with the second lag positively set the pace for the signature trail. Again, the first lag in the PACF correlogram (Figure 4B) is predominant. Thus, a differencing step may be also an advisable action, in disagreement with the augmented Dickey–Fuller test outcomes (Table 2). The KPSS test outcome hints at a trend-stationary condition. It is interesting that the serial temperature profile #4 was barely found to be nonstationary according to the augmented Dickey–Fuller test outcomes (Table 2). 

The corresponding correlograms of ACF (Figure 3D) and PACF (Figure 4D) seem to confirm this premise and differencing could aid in revealing additional trends. On the other hand, the temperature serial profile #5 exhibits a pattern (Figure 3E and Figure 4E) that could be benefited by a direct addition of an AR term; it agrees with the augmented Dickey–Fuller test outcomes (Table 2) that the time series is stationary. Overall, it may be said that no sequence signature could be deemed as purely “white noise” (no autocorrelation) based on the ARIMA results. 

**Figure 5:**
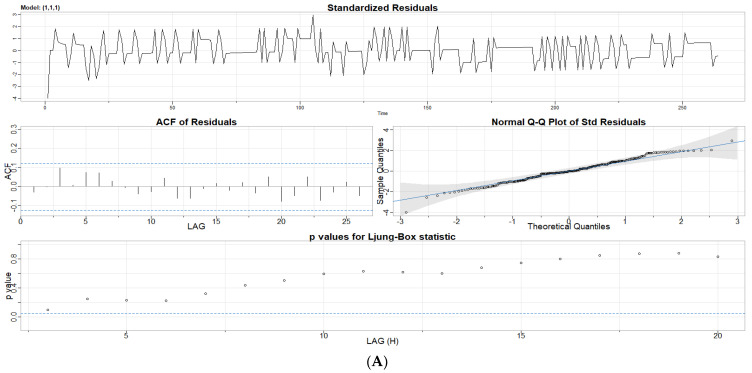

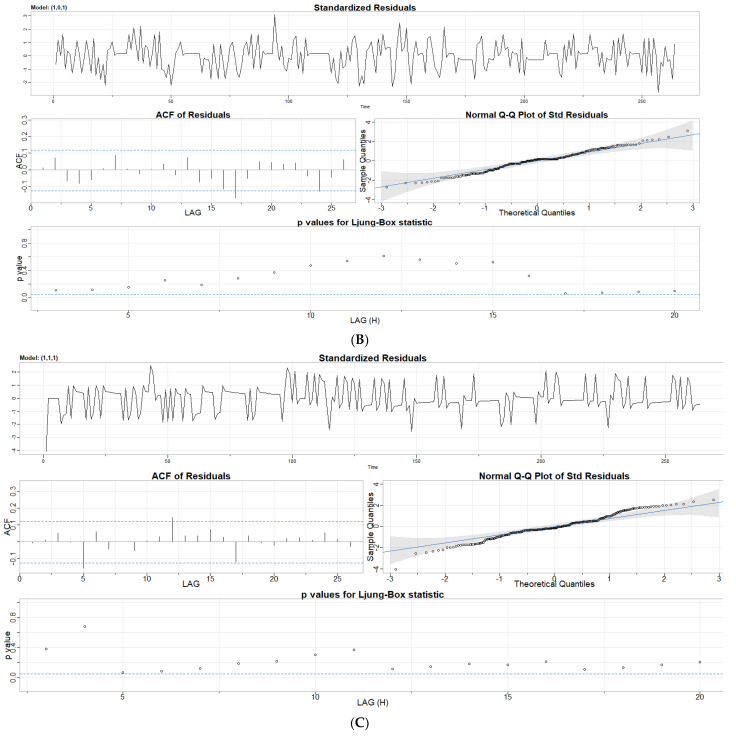

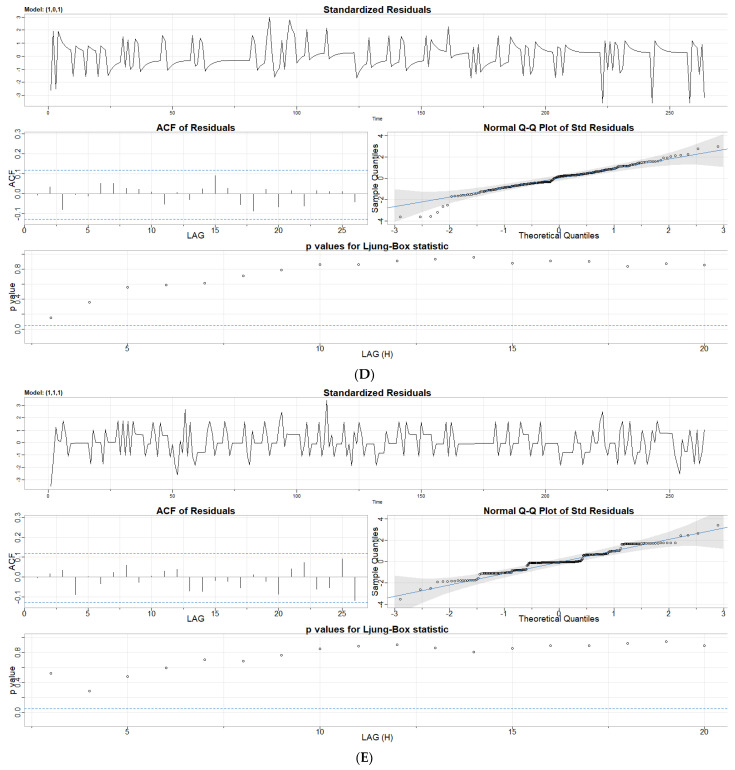

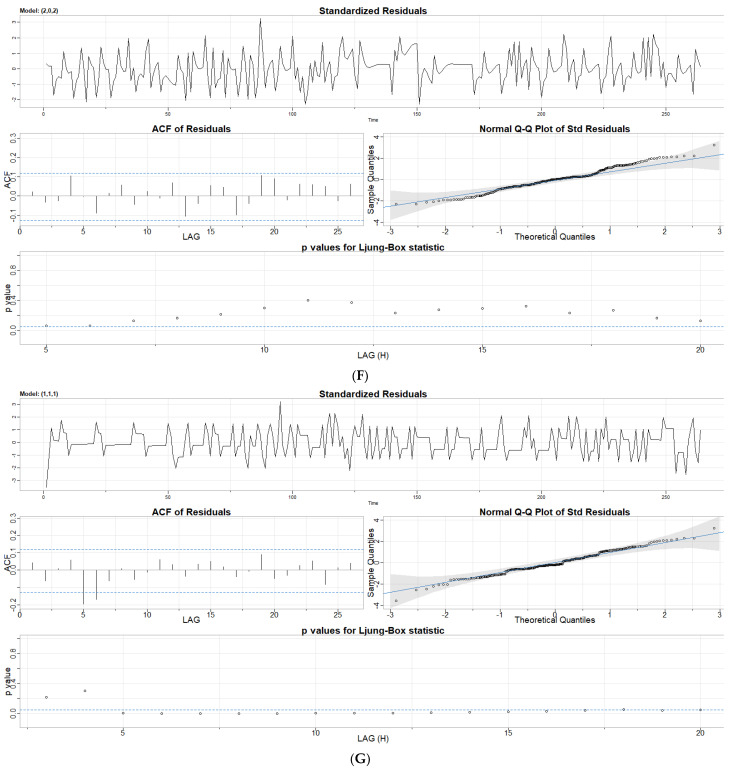

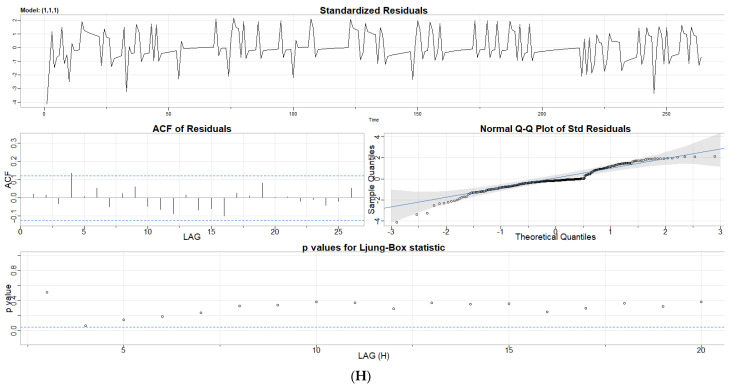
ARIMA modeling diagnostics for the eight temperature time series: (**A**–**H**) residuals plots.

Even more surprisingly, none of the time series needed to be adjusted with MA terms. The ARIMA analysis concludes with an assorted residual analysis to the fitted AR and MA coefficients for each individual temperature data sequence (Table 4). It is a common occurrence that, while there is a great variation in the sign and magnitude of the estimated values among the fitted AR and MA coefficients, seven out of the eight time series could be at least tentatively described by the ARIMA (1,1,1) model. Time series #6 required a higher order of AR and MA terms with no differencing (ARIMA (2,0,2)). The residual analysis results that accompany the model fittings are shown in Figure 5. With the exception of time series #7, the temperature data sequences comply with the minimum requirements for randomness of errors in view of their witnessed patterns of their standardized residuals sequences. Despite some portrayals of the residual ACFs, to not quite reducing to a strong “white noise” profile, diagnostics from the normal Q-Q plots are more supportive of the residuals randomness hypothesis. The Ljung–Box test [71] results assert that the profiled signatures follow a consistent randomness pattern in all seven error sequences (portmanteau test), including the diagnostics of time series #6 data. Temperature temporal sample #7 failed the Ljung–Box test as its data points are not independently distributed. It displays serial correlation, even though the Q-Q plot depiction appears to be more affirming on the sample randomness (Figure 5G). It is intriguing that the Akaike Information Criterion values for all ARIMA models are comparable (Table 4). Furthermore, AIC and BIC evaluations of the models are also analogous. 

### 3.2. Time Series Mode Screening Using Continuous Time Quantum Random Walks

**Figure 6:**
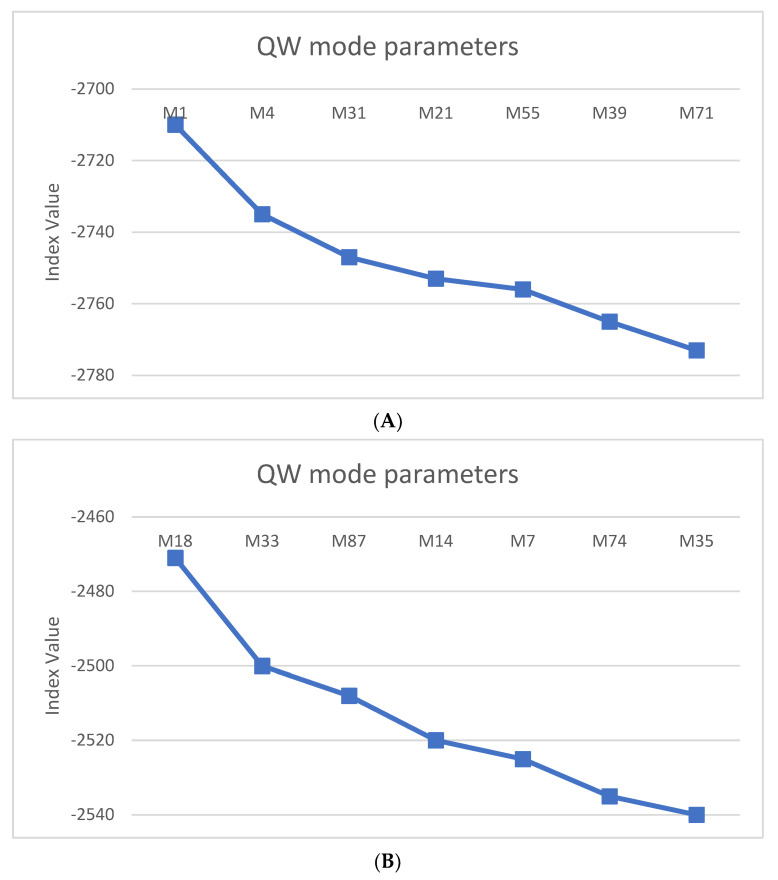

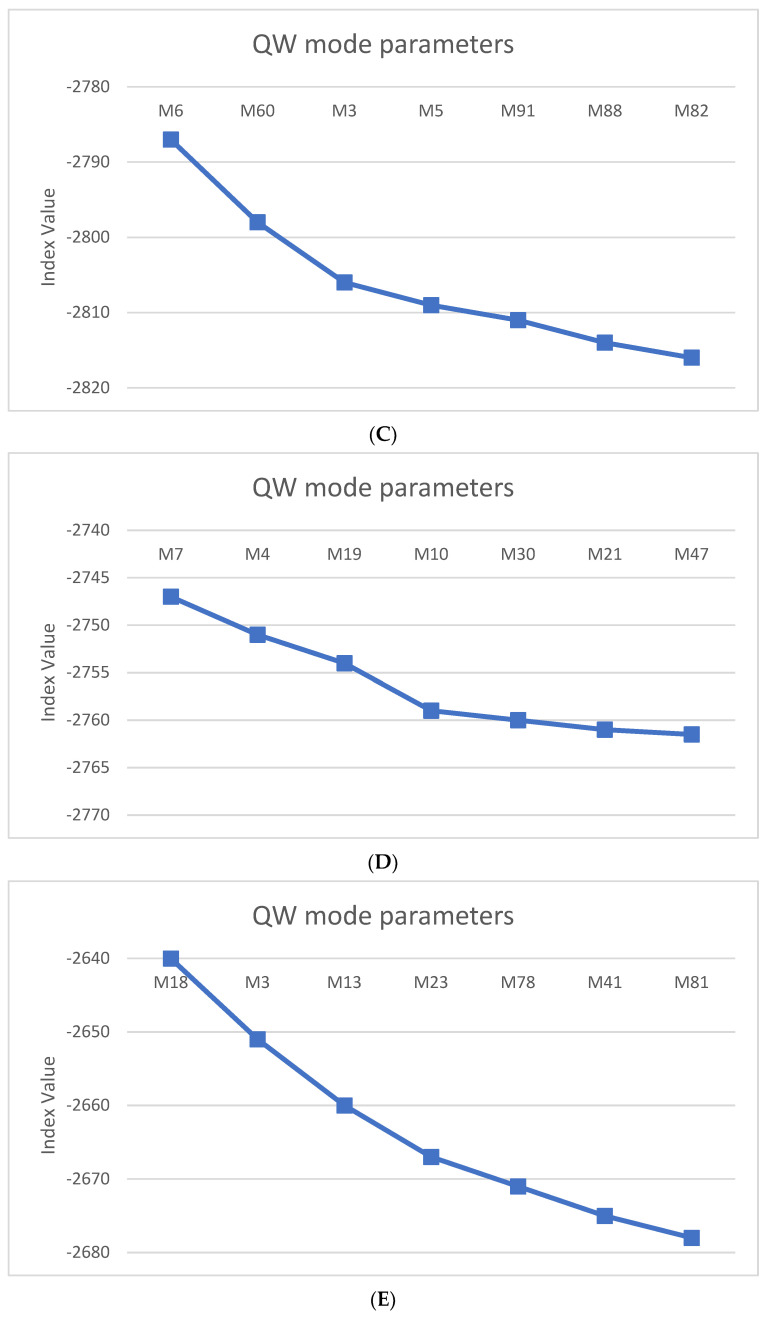

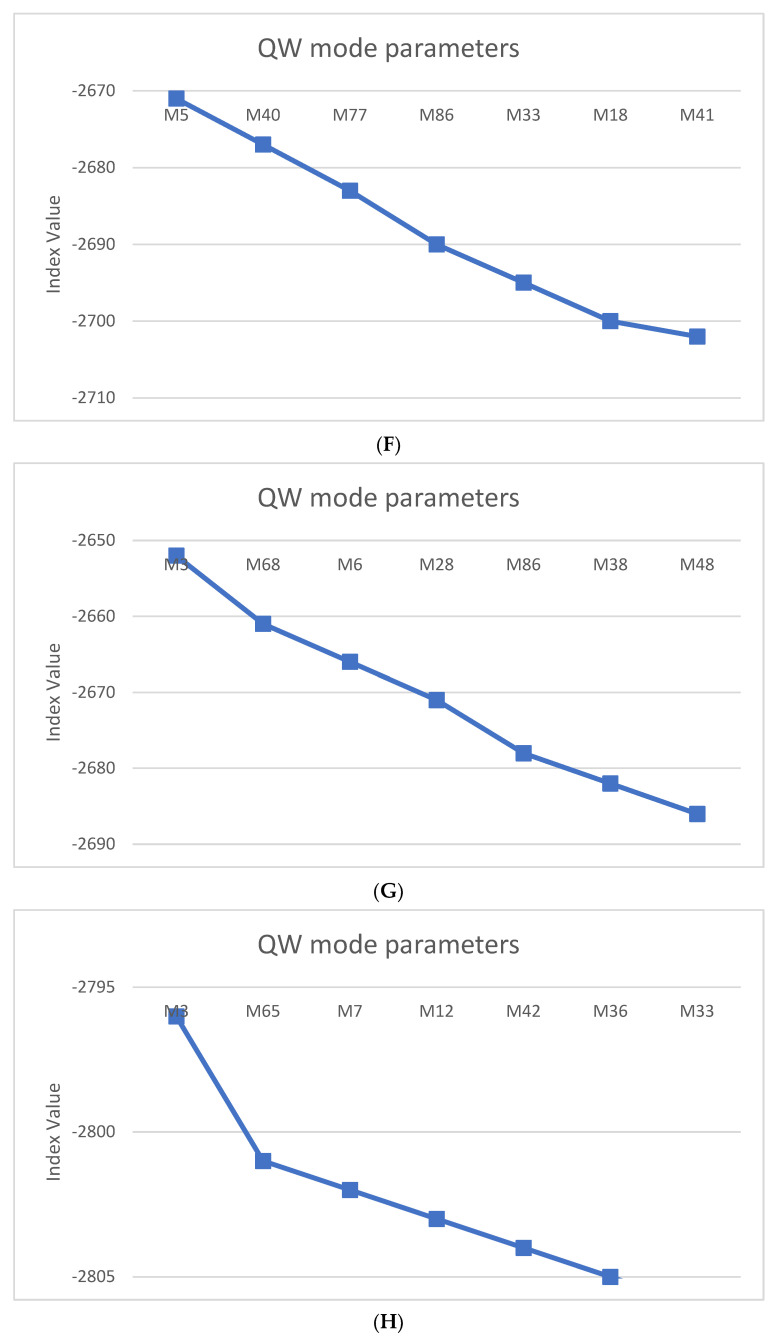
Scree-type plots (the quantum walker index value) for the eight temperature time series with increasing sensor identification numbers: (**A**–**H**) temperature plots.

**Figure 7:**
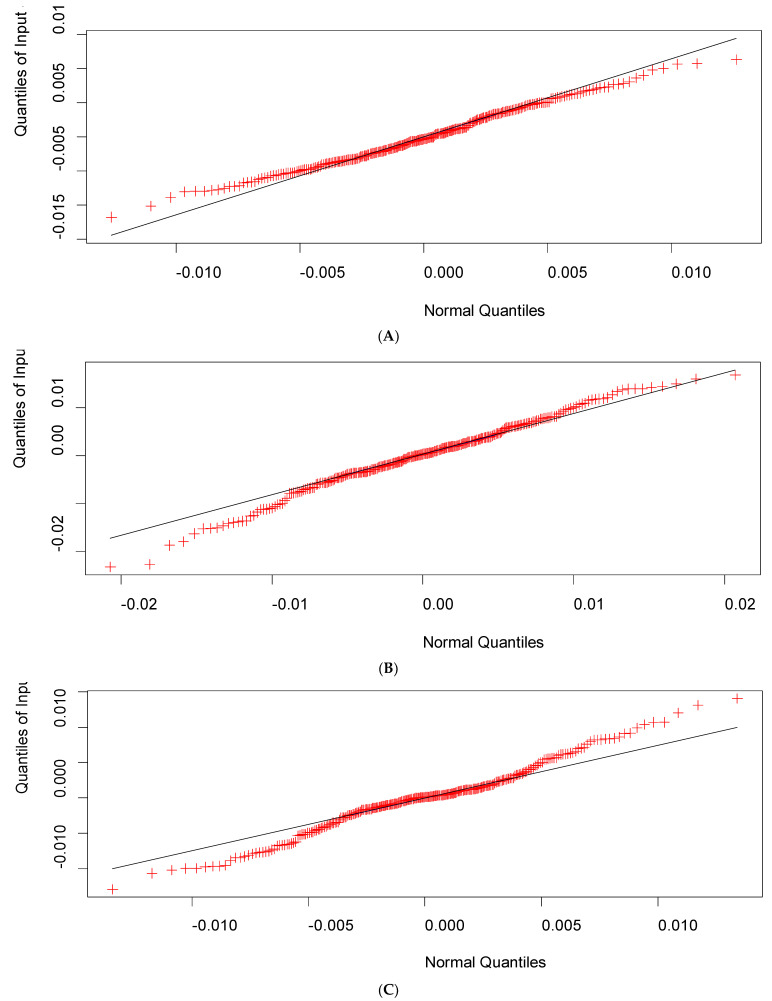

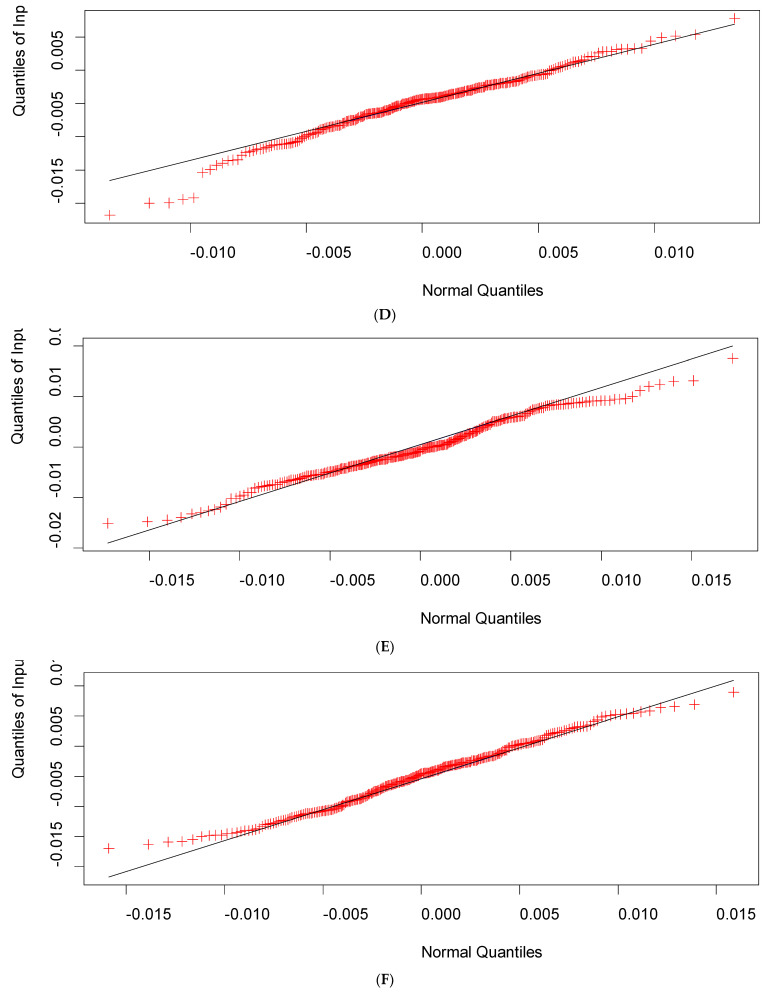

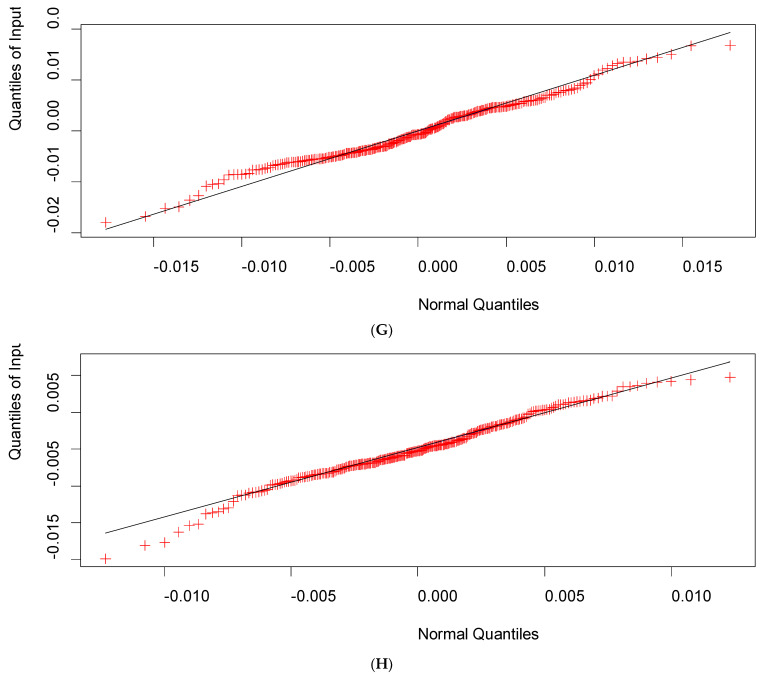
Q-Q plots (the quantum walker regression coefficients) for the eight temperature time series with increasing sensor identification numbers: (**A**–**H**) temperature plots.

In Figure 6, the eight scree plots of the index value for the quantum walker solution are depicted as they were computed by the R-package ‘QWDAP’. The decision to extract and retain certain non-trivial modes is automatically accomplished by the algorithm. It is primarily on a single “elbow” point that the cutoff is applied. However, it might be considered that for time series #3, 5 and 8, it perhaps included mode information from multiple elbow points. In Figure 7, the Q-Q plot technique is used to provide a graphical summary of the prediction performance of the residual analysis on the quantum walk/regression results shown in Figure 6. The quantum walker performance on fitting regressed coefficients for the temporal sequences #3, 4, 5, 6, 7, and 8 was satisfactory. Low-end quantiles in the fitted plots of time series #1 and 2 (Figure 7A, B) may not be so well compensated, though. 

In Table 5, the retained modes from the scree plot screenings are tallied and listed. It is obvious that there is a great variation among the total number of non-trivial modes as they range from a low value of 7 to a high value of 22. Its coefficient of variation value is calculated to be 0.38, which indicates that there is a substantial dispersion in the retained modes among different fitted time series. The mean and the standard deviation values were estimated at 12.1 (median value of 11) and 4.5 (interquartile range value of 3.5), respectively. This may be interpreted as an indication of non-uniform measuring performance at the eight sensor locations. It offers a quick glimpse of the unstable capability outlook, even when lacking other, more formal, statistical inference measures. The scree plot with the least non-trivial modes (serial sample #7) is shown in Figure 6D, where a maximum curvature (“elbow” point) is well formed; it may be used for comparing mode identification sequences across other time series in order to pinpoint prime sources of influence. On the other hand, in Figure 6B (serial sample #2), the time series requires more than three times the number of modes to capture the temperature fluctuations associated with sensor unit #2. Based on this observation, the seven modes with the highest contributions are tabulated in Table 5, in the form of pseudo-ordered sequences (from left-to-right). 

There are no sequences that are either filled completely by the same modes or on the same location on their ordered sequences. Symptomatically, from a total of 100 assigned modes, mode #3 appears as a leading contributor in three serial temperature datasets: #1, 7, and 8. It also contributes to the retained list that fits time series #3 and 5, as they are placed third and second on the corresponding ordered sequences, respectively. Mode #7 contributes to three time series (#2, 4, and 11). Similarly, mode #33 influences data sequences #2, 6, and 8. Finally, in Table 5, the estimated values of the Akaike Information Criterion (AIC) for the entire temperature time series dataset are tightly grouped since their calculated coefficients of variation value is quite low (0.033), given that the estimated mean and standard deviation values were 1781.9 and 58.3, respectively. The median value is 1788 and its associated interquartile range value is merely 74. This reflects the computational consistency achieved by the quantum-walk solver to extract most of the information from dissimilar data sequences. Notably, it may be said that in, searching for quantifying instabilities at a temperature end-scale point of a calibration study, the adoption of a quantum walker algorithm may aid in more quickly reaching a robust result for another crucial reason, besides the superb properties it possesses in that direction owing to the fundamental nature-based stochastic framework it represents (Section 2.1.2). A quantum walker algorithm is not burdened by the additional optimization work that is required to fine tune the hyper-parameters of simpler machine learning techniques such as tree-based ensample learning as well as a wider range of deep learning algorithms. 

## 4. Conclusions

Calibrating a thermostatic bath may be confronted with several issues when uniformity and stability considerations must be taken in account. Particularly challenging which arise may include situations where an ensemble of sensors is allocated to a bath fluid to be tested at the boundary of their specified temperature ranges. The evaluations of stability and uniformity of the measurements is modulated by several factors that may, randomly or not, interfere with the accuracy and resolution capabilities of the deployed group of sensors, accruing unexplainable contributions to the screening uncertainty. Based on an eight-sensor ensemble prepared to test the uniformity and stability properties of a thermostatic bath, temperature measurements were collected in parallel and stored as eight time series, at a limiting temperature of −20 °C. The selected sensor configuration was adapted to a published electronic thermometer verification scheme. A classical data analysis was carried out to examine data normality, serial stationarity, and to inspect the time-series data pattern for independent and identical distribution. AR and MA contributions were considered during the calibration modelling to find out whether the eight time-series datasets might exhibit a common-basis behavior at the chosen measuring locations. Overall, it was found that the eight time series were non-normally distributed, even though they might manifest a stationary evolution. Several statistical methods were employed in parallel to affirm the displayed unstable tendencies in the datasets. It was realized that using a quantum walker algorithm aided in accelerating the diagnosis of any observed uniformity and stability deviations. Conclusions were reached by merely establishing the number and the order of the fitted coefficients, which are related to the network (node-to-node) movement of a quantum walker. This was accomplished by individually tracking the probability evolution of the quantum pathfinder on each of the eight temperature data streaks. The significant reduction of the total amount of data processing is greatly appreciated since the monitored (multiple-location) temperature fluctuations would otherwise undergo an exhaustive stochastic analysis, which incorporates information ranging from the control-based time-series modelling to the statistical inference using well-accepted hypothesis-testing treatments with assorted graphical support. A key limitation of the developments in this work is that a good grasp of the inner workings of quantum theory may be required in order to successfully apply such state-of-the art nature-based stochastic algorithms. This may be crucial for some measurement environments with the aim of properly interpreting the findings. Perhaps incorporating such deep knowledge in more engineering disciplines will facilitate their early and speedy adoption. This work could be extended to test calibration behaviors among different sensor arrangements as well as different end-specification temperatures and various thermostatic bath fluids.

## Figures and Tables

**Table 1:** Testing the eight time series for normality using three statistical tests.

	AD-Test ^1^	SW-Test ^2^	JB-Test ^3^
**Sensor Time Series ID #**	A_n_ (*p*-Value)	W (*p*-Value)	X^2^ (*p*-Value)
**1**	53,183 (*p* < 0.001)	0.797 (*p* < 0.001)	1.77 (0.411)
**2**	53,774 (*p* < 0.001)	0.908 (*p* < 0.001)	1.73 (0.420)
**3**	53,822 (*p* < 0.001)	0.711 (*p* < 0.001)	2.97 (0.226)
**4**	53,313 (*p* < 0.001)	0.753 (*p* < 0.001)	3.65 (0.161)
**5**	53,491 (*p* < 0.001)	0.828 (*p* < 0.001)	0.64 (0.727)
**6**	53,617 (*p* < 0.001)	0.792 (*p* < 0.001)	1.14 (0.565)
**7**	53,633 (*p* < 0.001)	0.799 (*p* < 0.001)	6.50 (0.039)
**8**	53,446 (*p* < 0.001)	0.741 (*p* < 0.001)	3.62 (0.164)

^1^ Anderson–Darling; ^2^ Shapiro–Wilk; ^3^ Jarque–Bera.

**Table 2:** Testing the eight time series for stationarity using two statistical tests.

	Augmented Dickey-Fuller Test	KPSS Test	Maximum Drawdown (°C)
**Sensor Time Series ID #**	DF (*p*-Value)	KPSS (*p*-Value)	
**1**	−4.510 (*p* < 0.01)	1.209 (*p* < 0.01)	0.03
**2**	−4.572 (*p* < 0.01)	0.027 (*p* > 0.1)	0.05
**3**	−5.376 (*p* < 0.01)	0.739 (*p* < 0.01)	0.02
**4**	−3.419 (*p* = 0.052)	0.302 (*p* > 0.1)	0.03
**5**	−4.874 (*p* < 0.01)	0.252 (*p* > 0.1)	0.04
**6**	−6.819 (*p* < 0.01)	0.368 (*p* = 0.091)	0.03
**7**	−7.031 (*p* < 0.01)	0.981 (*p* < 0.01)	0.03
**8**	−4.146 (*p* < 0.01)	0.807 (*p* < 0.01)	0.03

**Table 3:** Runs test and BDS test results to examine independence and identical distribution of the eight time series.

		Runs Test			BDS Test
Temperature Sensor ID #	Median (°C)	Runs	*p*-Value	Epsilon Values	*p*-Value
**1**	−19.93	47	*p* < 0.001	0.003	*p* < 0.001
				0.007	*p* < 0.001
				0.010	*p* < 0.001
				0.013	*p* < 0.001
**2**	−20.04	35	*p* < 0.001	0.005	*p* < 0.001
				0.010	*p* < 0.001
				0.015	*p* < 0.001
				0.020	*p* < 0.001
**3**	−20.05	55	*p* = 0.004	0.003	*p* = 0.334
				0.005	*p* = 0.334
				0.008	*p* = 0.334
				0.011	*p* < 0.001
**4**	−19.95	11	*p* = 0.009	0.003	*p* < 0.001
				0.006	*p* < 0.001
				0.009	*p* < 0.001
				0.012	*p* < 0.001
**5**	−19.99	65	*p* < 0.001	0.004	*p* < 0.001
				0.007	*p* < 0.001
				0.011	*p* < 0.001
				0.014	*p* < 0.001
**6**	−20.01	31	*p* < 0.001	0.003	*p* < 0.001
				0.006	*p* < 0.001
				0.010	*p* < 0.001
				0.013	*p* < 0.001
**7**	−20.02	78	*p* < 0.001	0.003	*p* < 0.001
				0.007	*p* < 0.001
				0.010	*p* < 0.001
				0.013	*p* < 0.001
**8**	−19.98	54	*p* < 0.001	0.003	*p* < 0.001
				0.006	*p* < 0.001
				0.009	*p* < 0.001
				0.011	*p* < 0.001

**Table 4:** Autoregressive and moving average coefficient statistics (fitted ARIMA model) for the eight temperature data sequences.

	AR Coefficient Statistics				MA Coefficient Statistics					
Temperature Sensor ID #	Estimate	Standard Error	t-Value	*p*-Value	Estimate	Standard Error	t-Value	*p*-Value	AIC	BIC
**1**	0.431	0.056	7.692	*p* < 0.001	−1.000	0.019	−52.025	*p* < 0.001	−7.70	−7.64
**2**	0.643	0.061	10.477	*p* < 0.001	0.145	0.074	1.956	*p* = 0.052	−7.10	−7.04
**3**	0.183	0.067	2.746	*p* = 0.007	−0.944	0.025	−37.962	*p* < 0.001	−7.75	−7.70
**4**	0.897	0.046	19.437	*p* < 0.001	−0.663	0.080	−8.346	*p* < 0.001	−7.65	−7.60
**5**	0.567	0.051	11.086	*p* < 0.001	−1.000	0.014	−69.782	*p* < 0.001	−7.44	−7.39
**6**	1.455 −0.718	0.092 0.081	15.757 −8.851	*p* < 0.001 *p* < 0.001	−0.967 0.361	0.118 0.120	−8.173 3.002	*p* < 0.001 *p* = 0.003	−7.65	−7.57
**7**	0.465	0.055	8.482	*p* < 0.001	−1.000	0.0102	−97.811	*p* < 0.001	−7.47	−7.41
**8**	0.242	0.070	3.461	*p* < 0.001	−0.930	0.030	−30.737	*p* < 0.001	−7.79	−7.74

**Table 5:** Retained number of quantum walker (regression) modes of the eight temperature time series.

Temperature Sensor ID	Retained no. of Modes	Leading Mode Sequences	AIC
**1**	14	3-4-31-21-55-39-71	1725
**2**	22	18-33-87-14-7-74-35	1691
**3**	9	6-60-3-5-91-88-82	1850
**4**	7	7-4-19-10-30-21-47	1858
**5**	11	18-3-13-23-78-41-81	1780
**6**	10	5-40-77-86-33-18-41	1806
**7**	13	3-68-6-28-86-38-48	1749
**8**	11	3-65-7-12-42-36-33	1796

## Data Availability

The original data are available through Mrs. Valeli’s MSc thesis as submitted to the Kingston University depository per ref. [58].
